# Time series analysis of cumulative incidences of typhoid and paratyphoid fevers in China using both Grey and SARIMA models

**DOI:** 10.1371/journal.pone.0241217

**Published:** 2020-10-28

**Authors:** Jiaqi Gao, Jiayuan Li, Mengqiao Wang

**Affiliations:** Department of Epidemiology and Biostatistics, West China School of Public Health and West China Fourth Hospital, Sichuan University, Chengdu, P. R. China; The Chinese University of Hong Kong, HONG KONG

## Abstract

Typhoid and paratyphoid fevers are common enteric diseases causing disability and death in China. Incidence data of typhoid and paratyphoid between 2004 and 2016 in China were analyzed descriptively to explore the epidemiological features such as age-specific and geographical distribution. Cumulative incidence of both fevers displayed significant decrease nationally, displaying a drop of 73.9% for typhoid and 86.6% for paratyphoid in 2016 compared to 2004. Cumulative incidence fell in all age subgroups and the 0–4 years-old children were the most susceptible ones in recent years. A cluster of three southwestern provinces (Yunnan, Guizhou, and Guangxi) were the top high-incidence regions. Grey model GM (1,1) and seasonal autoregressive integrated moving average (SARIMA) model were employed to extract the long-term trends of the diseases. Annual cumulative incidence for typhoid and paratyphoid were formulated by GM (1,1) as $\hat{x}(t)=-14.98(e^{-0.10(t-2004)}-e^{-0.10(t-2005)})$ and $\hat{x}(t)=-4.96(e^{-0.19(t-2004)}-e^{-0.19(t-2005)})$ respectively. SARIMA (0,1,7) × (1,0,1)_12_ was selected among a collection of constructed models for high R^2^ and low errors. The predictive models for both fevers forecasted cumulative incidence to continue the slightly downward trend and maintain the cyclical seasonality in near future years. Such data-driven insights are informative and actionable for the prevention and control of typhoid and paratyphoid fevers as serious infectious diseases.

## Introduction

Typhoid and paratyphoid fevers, collectively referred to as enteric fever, are caused by systematic infection with the gram-negative bacterium *Salmonella enterica* serotype *S*. *typhi* and *S*. *paratyphi* (types of A, B, and C) [1]. The organisms enter the patients via the gastrointestinal tract and get into the bloodstream via the lymphatic channels, and a mouse model has been engineered [2]. Sanitary measures and personal hygiene play instrumental role as infections generally occur after intaking food or water contaminated by urine or feces [3]. The incubation period could generally last from 3 to 42 days, with on average 14 days for typhoid and 2–15 days for paratyphoid [4]. Clinical manifestations include high-temperature fever, prostration, fatigue, headache, and gastrointestinal reactions, with serious complications such as intestinal bleeding and perforation [5]. With symptoms not exclusive compared to other types of fevers, diagnosis of both typhoid and paratyphoid is conducted through clinical culture and test of patients’ blood, stool, or urine. Live-attenuated oral vaccine or capsular polysaccharide vaccine are currently available for prevention, and treatment options include ceftriaxone, ciprofloxacin, or azithromycin.

Both types of fevers are more common in developing than developed countries. In south Asia, southeast Asia, and sub-Saharan Africa areas with poor water supply and sanitation, they are a major cause of death and disability, especially among children, and have a significant impact on social and economic development [6–8]. Environmental factors, such as climate, have also been investigated to assess their influence on water-food-borne infections [9–12]. According to the estimate by the World Health Organization (WHO) in 2010, nearly 22 million cases of typhoid fever occur annually, with at least 200,000 deaths [10]. China has been among the high-incidence areas, with cumulative incidence about 10–50 per 100,000 before 1990 but gradually decreasing since then [13]. Nevertheless, typhoid and paratyphoid fevers remain important sporadic intestinal infectious diseases and are directly monitored nationwide. Descriptive epidemiology research has been conducted on specific provinces [14–17], but there remains a lack of national analysis on time series modeling which is key for the prevention and control measures [18]. Several mathematical models have been employed to predict the incidence of infectious diseases [19]. Based on the window size of the independent variable, the grey model (GM) predicts future value of the time series using only the most recent set of data. The GM assumes all data are positive (consistent with cumulative incidence) and sampling frequency of the time series is fixed (true for annual or monthly datasets). The grey model does not require errors in normal distribution and is hardly limited by small sample size [20]. Seasonal Autoregressive Integrated Moving Average (SARIMA) model, simultaneously taking general trend, periodic pattern, and random disturbance into consideration, has been widely applied in the research on various infectious diseases with periodic pattern, e.g. malaria [21, 22], hand-foot-mouth disease [23], AIDS [24], and tuberculosis [25]. Due to the infectivity and seasonality of infectious disease, SARIMA has more predictive power compared to other mathematical models [26, 27].

This study aims to conduct a thorough analysis of the long-term cumulative incidence of both typhoid and paratyphoid fevers in China at both national and regional levels, at both yearly and seasonal windows, and on the susceptibility of people of various ages. Statistical models of GM (1,1) and SARIMA would also be applied to fit historic yearly and monthly data for revealing the underlying structure of the infectious trends, and to forecast future incidence to provide data-driven knowledge for the prevention and control of these fevers.

## Methods

### Data sources

Typhoid or paratyphoid fevers were categorized as type-II infectious diseases by Centers for Disease Control and Prevention of China, and the historic incidence data from 2004 to 2016 were retrieved from the portal of Chinese Public Health Science Database (CPHSD, http://www.phsciencedata.cn/). Detailed data for 2017 and 2018 were not yet published in the database, but annual incidence for the combined fevers in both years was reported by official government news releases. Regional data covered 31 provincial regions in mainland China. All patients were diagnosed using criteria (GB 16001–1995) promulgated by the Ministry of Health of China: the patient had persistent fever (higher than 40°C) of unknown origin, accompanied by positive serum antibody (titer ≥ 1:80 by the Widal test, agglutination titer of typhoid or paratyphoid flagella ≥ 1:160), or the patient had unexplained fever, and *S*. *typhi* or *S*. *paratyphi* could be isolated in any specimen of serum, bone marrow, feces, or bile.

### Time series models

GM (1,1) model—for GM (*n*, *m*), *n* is the order of the differential equation and *m* is the number of variables. GM (1,1) model can be used to predict incomplete or uncertain data. With original time series data *x*^(0)^(*t*) = (*x*^(0)^(1),*x*^(0)^(2),*x*^(0)^(3),…,*x*^(0)^(*n*)), define cumulative sum of $y(t)=\sum_{i=1}^{t}x^{\left(0\right)}(t),\;t=1,2,3,\ldots ,n$. The adjacent mean is z(t) = (*y*(*t*)+*y*(*t*−1))/2.

With white-formed ordinary differential equation, D, a and u are estimated by the equations as follow:

$$D=(n-1)[\sum_{t=2}^{n}z^{2}(t)]+[\sum_{t=2}^{n}z(t){]}^{2}$$ (1)

$$a=\{(n-1)[-\sum_{t=2}^{n}x^{\left(0\right)}(t)z(t)]+[\sum_{t=2}^{n}z(t)][\sum_{t=2}^{n}x(t)]\}/D$$ (2)

$$u=\{[\sum_{t=2}^{n}z(t)][-\sum_{t=2}^{n}x^{\left(0\right)}(t)z(t)]+[\sum_{t=2}^{n}z^{2}(t)][\sum_{t=2}^{n}x(t)]\}/D$$ (3)

Substitute the values of a and u into the formula: $\hat{y}(t)=[x^{\left(0\right)}(1)-\frac{u}{a}]e^{-a(t-1)}+\frac{u}{a},$ then the estimated value of *x*^(1)^(*t*) can be calculated as $x^{\left(1\right)}(t)=\hat{y}(t)-\hat{y}(t-1)$. The grey model can be used for extrapolative prediction only after passing the accuracy test of small error probability and posterior error ratio.

SARIMA model—in SARIMA(p, d, q) (P, D, Q)_s_, p, d, and q annotate the order of auto-regression, the degree of trend difference, and the order of moving average respectively; P, D, and Q represent the seasonal auto-regression lag, the degree of seasonal difference, and the seasonal moving average; s annotates the length of the cyclical pattern. In SARIMA model fitting, the Augmented Dickey-Fuller (ADF) unit-root test evaluated the stationary status of time series; seasonal and non-seasonal differences were adopted to stabilize the term trend and periodicity. Parameters of SARIMA model were estimated by autocorrelation function graph and partial autocorrelation graph. Models of varying orders of p, q and P, Q were tested through Box-Jenkins test, and all models passing the residual test were evaluated for performance using criteria including mean absolute percentage error, root mean square error, Akaike Information Criterion (AIC), and R^2^. For SARIMA, data from 2004 to 2015 were used in model fitting, and data in 2016 were used as out-of-sample set for prediction validation.

### Data analysis

Data analysis and visualization were performed in version 4.0.0 of the R statistical software (R Core Team, 2020). Significance level of 0.05 is used in the null-hypothesis test unless otherwise specified.

## Results

### Typhoid and paratyphoid fevers in China

**Fig 1:**
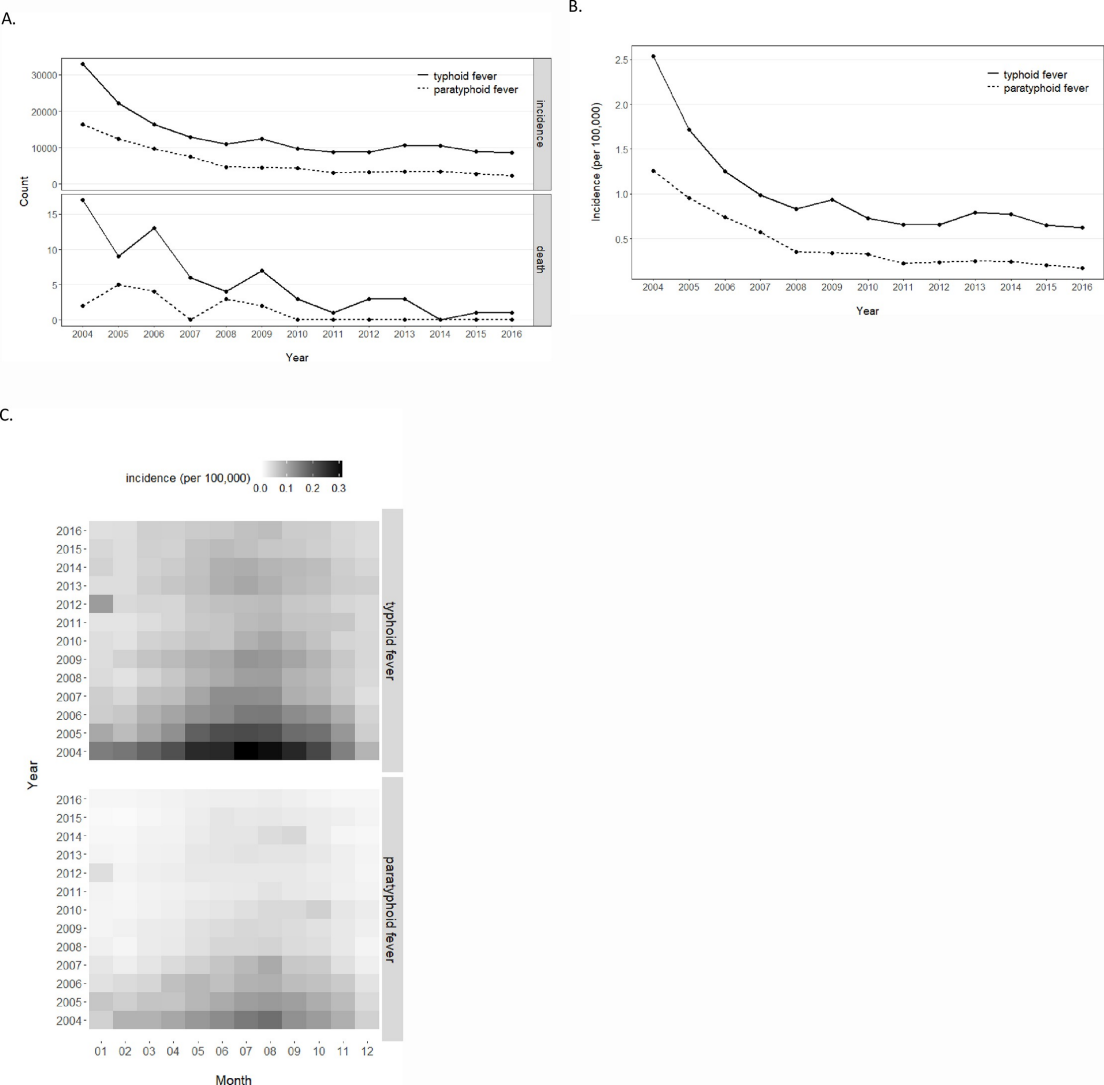
Annual incidence and death counts (A), annual cumulative incidence (B), and monthly cumulative incidences (C) of typhoid and paratyphoid fevers in China from 2004 to 2016. Note: sum of monthly incidence equals to the corresponding annual incidence.

Typhoid and paratyphoid fevers were by law monitored as type-II infectious diseases in China, and we tracked their recent long-term trends from 2004 to 2016 (Table 1). Nationally, incidence counts displayed a significant trend of decrease: almost 33000 cases of typhoid fever was reported in 2004 but the count fell below 10000 and stayed in the close range since 2010; more than 16000 cases of paratyphoid fever was reported in 2004, and the continual downfall reached to 2311 cases in 2016 (Fig 1A). With slightly expanding population during this period, the cumulative incidences mirrored and in fact better revealed the historic trends, observing a drop of 73.9% for typhoid fever and 86.6% for paratyphoid fever (Fig 1B). Cumulative incidence of typhoid fever was consistently higher (2–4 folds) than that of paratyphoid fever, and by 2016 they have respectively dropped to 0.63 and 0.17 cases per 100,000 population. Although the cumulative incidences were relatively low compared to other intestinal infectious diseases such as dysentery (9 cases per 100,000) and hepatitis (89 cases per 100,000), both types of fevers still appeared as infectious diseases of noticeable concern given their still high count of cases in the most populous country on earth. In contrast to the number of cases, deaths caused by both fevers were few (Fig 1A), and the very low mortality was different compared to other severe infectious diseases such as seasonal flu and severe acute respiratory syndrome (SARS). With characterization of cumulative incidence in months, both types of fevers displayed a clear seasonal pattern which peaked in the summer (mainly from June to August) and bottomed in the winter (interestingly, there appeared an “outlier” outbreak of both fevers in January of 2012) (Fig 1C).

**Table 1:** Statistics of typhoid and paratyphoid fevers in China, 2004 to 2016.

Year	typhoid		paratyphoid		combined	
	cases	incidence	cases	incidence	cases	incidence
2004	32966	2.54	16366	1.26	49332	3.80
2005	22270	1.71	12426	0.96	34696	2.67
2006	16317	1.25	9669	0.74	25986	1.99
2007	12942	0.98	7486	0.57	20428	1.55
2008	10946	0.83	4695	0.36	15641	1.18
2009	12427	0.94	4511	0.34	16938	1.28
2010	9716	0.73	4325	0.32	14041	1.05
2011	8768	0.65	3030	0.23	11798	0.88
2012	8816	0.65	3182	0.24	11998	0.89
2013	10722	0.79	3414	0.25	14136	1.04
2014	10460	0.77	3308	0.24	13768	1.02
2015	8844	0.65	2793	0.21	11637	0.85
2016	8588	0.63	2311	0.17	10899	0.80
2017*					10791	0.78
2018*					10843	0.78

Note: cumulative incidence as incidence per 100,000 people.

* data for 2017 and 2018 were not individually available in the public health database, but the combined data were reported by official government news releases.

### Age distribution of typhoid and paratyphoid fevers

**Fig 2:**
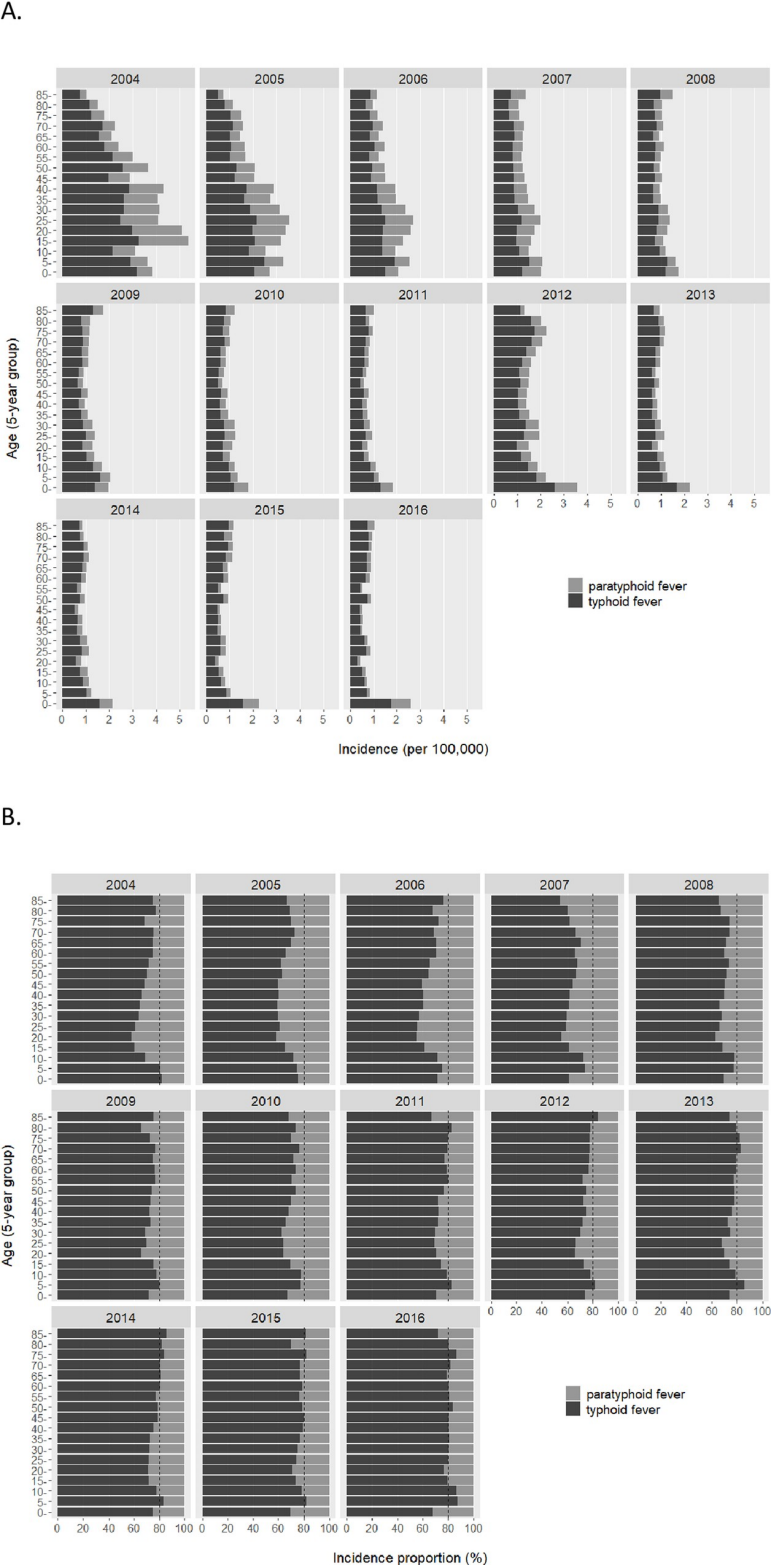
Age-specific annual cumulative incidence (A) and composition (B) of typhoid and paratyphoid fevers in China from 2004 to 2016.

People of different ages were substantially unalike with regards to physical, physiological, and psychological conditions, and consequently may be differentially susceptible to certain types of infectious diseases. In the earlier years of 2004 and 2005 when both typhoid and paratyphoid fevers were widespread, we observed higher cumulative incidence for young (15–29 years old) and middle-aged (30–44 years old) subgroups; however, as cases sequentially fell across all age groups throughout the following years, there no longer appeared a significant difference in cumulative incidence for different age groups except that the young children subgroup (0–4 years old) maintained the highest risk at double the level of other age groups (Fig 2A). Further analysis revealed that there was a quite consistent composition of typhoid fever and paratyphoid fever in 4:1 ratio across all age groups (more evident in recent than earlier year) (Fig 2B).

### Geographical cluster of typhoid and paratyphoid fevers

**Fig 3:**
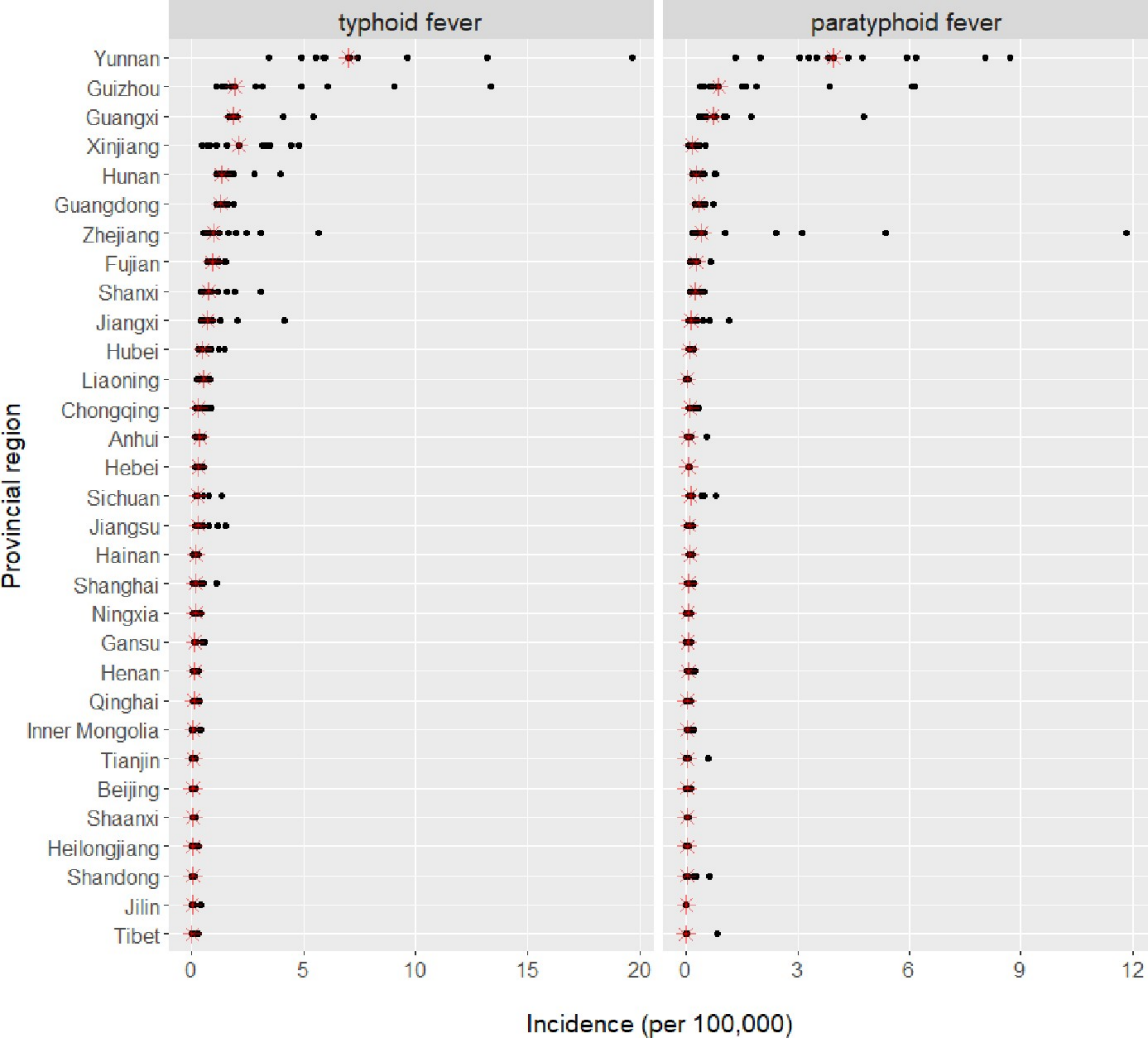
Region-specific annual cumulative incidence of typhoid and paratyphoid fevers in China from 2004 to 2016. Red marker annotates the median value. The regions are ordered by the median cumulative incidence of the combined typhoid and paratyphoid (sum of the red markers).

**Fig 4:**
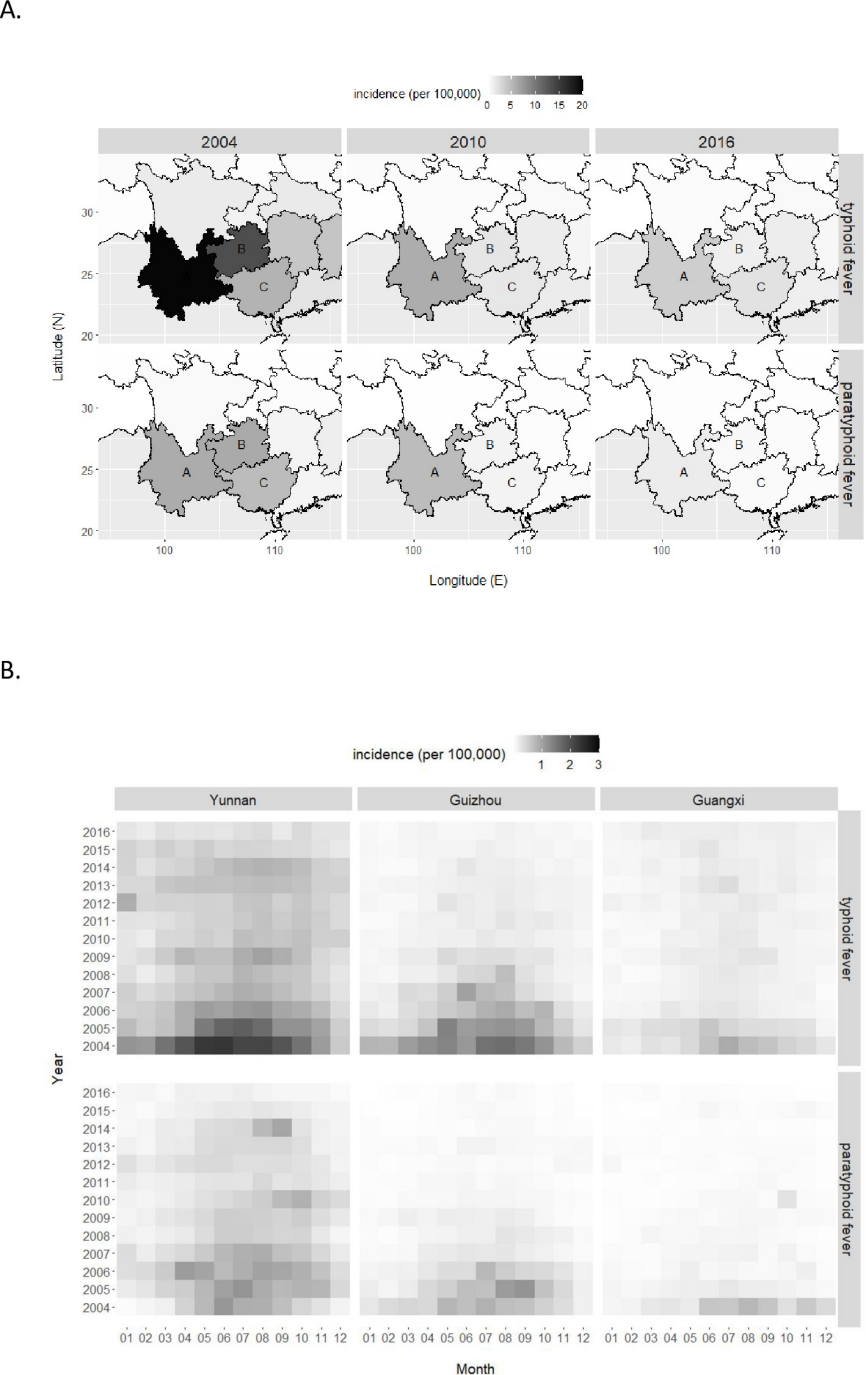
Annual (A) and monthly (B) cumulative incidences of typhoid and paratyphoid fevers in a cluster of three provinces (A. Yunnan, B. Guizhou, C. Guangxi).

National statistics in China generally covered 31 provincial regions, and accordingly we analyzed the annual cumulative incidence of typhoid and paratyphoid fevers by region. The sequential downward trend of cumulative incidence was observed universally in almost all provinces (S1 Fig). There also existed a geo-separation effect: cumulative incidences were clearly higher in southern provinces than northern provinces (Fig 3, S2 and S3 Figs). The top high-incidence provinces were Yunnan, Guizhou, and Guangxi (Fig 3), and interestingly, all three provinces were geographically adjacent and located to the southwestern part of China (bordering with Vietnam, Laos, and Myanmar). The top provinces also matched the similar decreasing trend and the seasonal pattern observed in the nation for both fevers (Fig 4A and 4B). Standing out from all others, Yunnan was the province of the highest cumulative incidence for both typhoid and paratyphoid fevers, at levels as 10–12 times high as national average and even 3–4 times high as the second highest province of Guizhou.

### Prediction of annual incidence by GM (1, 1)

**Fig 5:**
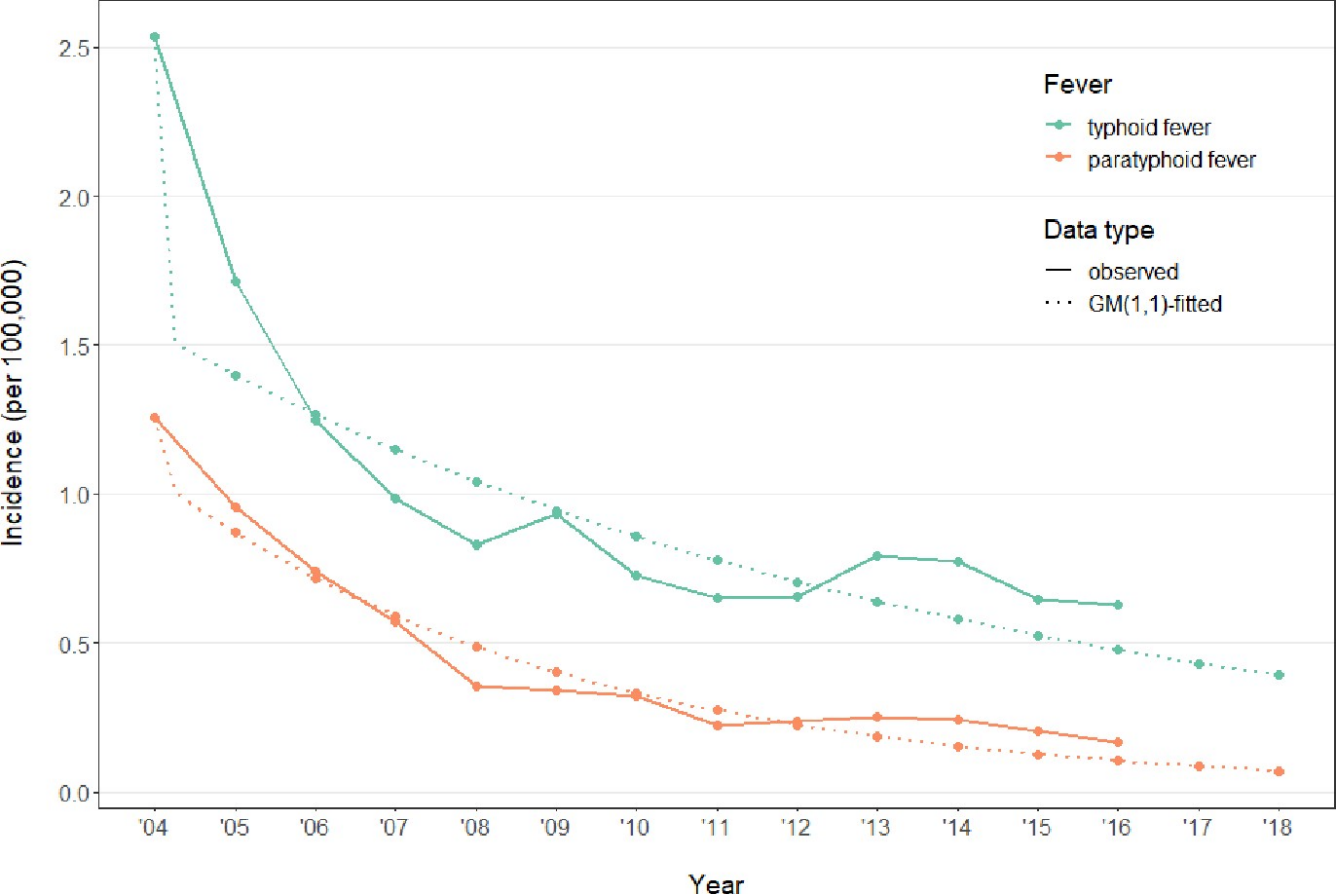
Annual cumulative incidences of typhoid and paratyphoid fevers as observed (solid line) and fitted by GM (1, 1) (dotted line).

GM (1,1), a basic type of grey model with first-order equation and single variable, was applied on both typhoid and paratyphoid fevers, and resulted in smooth exponential models that effectively tracked the downward trend of incidences (Fig 5). The GM (1,1) model for typhoid fever formulated cumulative incidence as $\hat{y}(t)=-14.98e^{-0.10(t-2004)}+17.51$ (with *t* annotating year) and thus modelled annual cumulative incidence (per 100,000) as $\hat{x}(t)=\hat{y}(t)-\hat{y}(t-1)=-14.98(e^{-0.10(t-2004)}-e^{-0.10(t-2005)})$ ($\hat{x}$(2004) at 2.54); similarly, the GM (1,1) model for paratyphoid fever formulated cumulative incidence as $\hat{y}(t)=-4.96e^{-0.19(t-2004)}+6.22$ and thus modelled annual cumulative incidence (per 100,000) as $\hat{x}(t)=\hat{y}(t)-\hat{y}(t-1)=-4.96(e^{-0.19(t-2004)}-e^{-0.19(t-2005)})$ ($\hat{x}$(2004) at baseline level at 1.26). Evaluation of the GM (1,1) performance revealed posterior error ratio at 0.17 and 0.12 respectively (both significantly smaller than the commonly used threshold of 0.35), and small error probability both higher than the commonly used criteria of 0.95 [26]. Therefore, the fitted models were statistically robust and thus may be applicable for the forecast of annual incidence in near future.

Indeed, the established grey models predicted cumulative incidence (per 100,000) in 2017 and 2018 at 0.43 and 0.39 for typhoid fever, and at 0.09 and 0.07 for paratyphoid fever (S1 Table). Most recent government news releases reported a combined “typhoid + paratyphoid” cumulative incidence of 0.78 in 2017 and 0.78 in 2018, which were close to the sum of our predicted incidence of 0.52 cases per 100,000 in 2017 and 0.46 cases per 100,000 in 2018. Admittedly, there appeared to be a slight underestimation of both fevers by the grey models for 2017 and 2018, which could be revealed by the fitting curve falling below the actual curve in years from 2013 to 2016 (Fig 5).

### Prediction of monthly incidence by SARIMA

**Fig 6:**
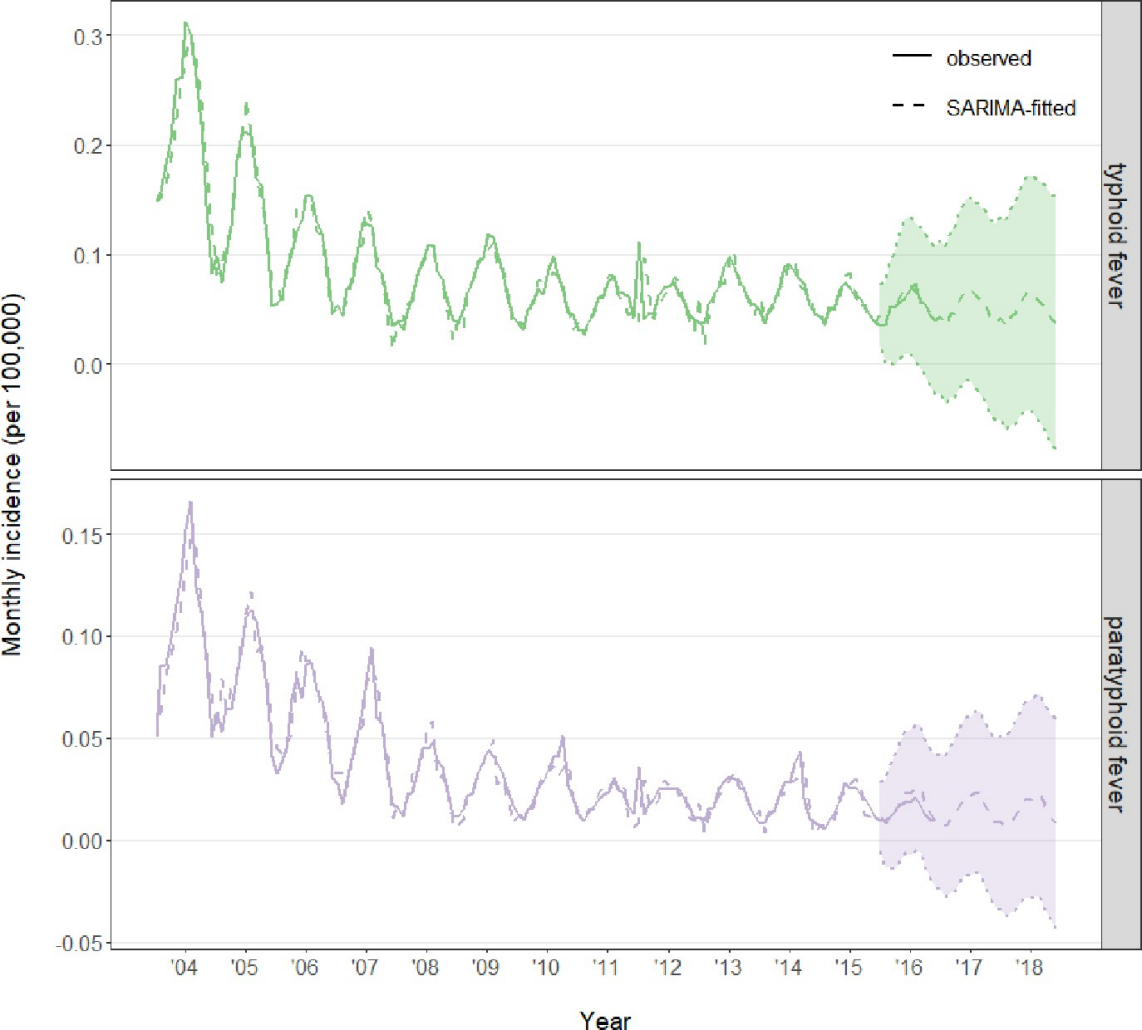
Monthly cumulative incidences of typhoid and paratyphoid fevers as observed (solid line) and fitted by SARIMA (0,1,7) × (1,0,1)_12_ (dashed line). A shaded 95% prediction interval was shown for 2016–2018.

Given the apparent seasonal pattern of monthly cumulative incidence, we conducted further time series analysis: a pseudo-forecast scenario was designed to use data from 2004 to 2015 for fitting SARIMA models and data in 2016 for validating model prediction (S2 Table). The incidence of both typhoid and paratyphoid fevers displayed apparent seasonal pattern (s = 12), and by a differential of non-seasonal (d = 1) and seasonal (D = 0) effects, the time series passed the ADF test (P < 0.05). A spectrum of parameter settings were compared in parallel in the goodness-of-fit test statistics, and an optimal model of SARIMA (0,1,7) × (1,0,1)_12_ was chosen given its top performance (high R^2^, low mean absolute percentage error, low AIC) for both typhoid and paratyphoid fevers (Table 2). Selection of the same SARIMA model also suggested similar seasonal and annual trends for both fevers. SARIMA (0,1,7) × (1,0,1)_12_ fitted the cumulative incidences well for the train set of 2004–2015 period, and even the “outlier” outbreak in January 2012 was uncovered; more importantly, for the independent validation set, SARIMA-fitted cumulative incidence closely matched the actual data in 2016, demonstrating the robustness of the predictive model (Fig 6 and S2 Table). For following years of 2017 and 2018, actual monthly incidence data were not yet published, but our SARIMA (0,1,7) × (1,0,1)_12_ model forecasted similar seasonal pattern for both fevers, which should be informative for the future prevention and control of these diseases (Fig 6).

**Table 2:** Goodness-of-fit summary of plausible SARIMA models.

Model	typhoid				paratyphoid			
	MAPE	RMSE	AIC	R^2^	MAPE	RMSE	AIC	R^2^
(2,1,2) × (1,1,1)_12_	14.536	0.015	-797.00	0.846	23.806	0.009	-861.19	0.854
(1,1,0) × (1,1,0)_12_	15.224	0.015	-797.03	0.836	21.226	0.010	-929.36	0.898
(1,1,0) × (1,1,2)_12_	15.368	0.015	-794.17	0.875	25.029	0.009	-859.21	0.854
(0,1,1) × (1,1,2)_12_	15.45	0.015	-793.73	0.834	25.239	0.009	-863.64	0.851
(1,1,0) × (1,1,2)_12_	15.553	0.015	-794.17	0.834	23.826	0.009	-867.39	0.854
(0,1,6) × (1,0,1)_12_	13.655	0.016	-865.24	0.916	20.014	0.010	-938.80	0.912
(0,1,7) × (1,0,1)_12_	13.257	0.016	-865.24	0.921	19.501	0.010	-937.30	0.914

Note: MAPE, mean absolute percentage error; RMSE, root mean square error; AIC, Akaike’s information criteria.

## Discussion

Typhoid and paratyphoid are associated with high infectiousness, large number of hidden carriers, and heavy disease burden. The trend in seasonality potentially suggested season-related factors (including but not limited to temperature, precipitation, humidity etc.) as key environment variables associated with these fevers [28–30]. Our analysis indicated that the incidence of typhoid and paratyphoid fevers continually decreased in China, and several factors potentially contributed to this well-demonstrated example of the prevention and control of infectious disease. First, higher living condition and better hygiene (water, food, toilet etc.) prevents the infectious pathways of the bacteria; second, available vaccination, though not mandatory but still recommended, provides extra protection to individuals particularly in those high-risk regions [16, 31]. People in all ages were susceptible to both diseases (typhoid fever presented higher cumulative incidence than paratyphoid fever), and the risk is particularly higher in the young children group, in agreement with findings from previous study [32].

While susceptibility of young children may be related to their lack of hygiene, an option not to receive the non-mandatory immunization, short-lasting immunity from the vaccines even when immunized, and disapproval of the vaccines for children under 2 years of age all contribute to the risk of typhoid and paratyphoid to this most vulnerable subgroup. As a countermeasure, the WHO in 2017 prequalified a new typhoid conjugate vaccine with longer lasting immunity for use in children from the age of 6 months, and the Strategic Advisory Group of Experts (SAGE) on immunization recommended such vaccines for routine use in children over 6 months of age in typhoid endemic countries [33].

The seasonal trends of typhoid and paratyphoid were apparent and consistent throughout years with higher incidence in the summer, implicating that intensive public health efforts should be applied in the spring and summer to contain any potential outbreak and epidemic. Geographical analysis demonstrated significant disparity in the incidence of typhoid and paratyphoid among provincial regions. The cluster in the southwestern region and particularly the hotspot of Yunnan potentially suggested that exclusive local features including geology, climate, and economy may contribute to the incidence of both fevers. The highest-incidence cluster in the southwestern part of China could be a result of pathogenic bacteria growing faster under high temperature and subtropical precipitation climate [34], and suboptimal status of economic development and public health condition likely leading to water pollution and food contamination [32, 35]. Further suppression of the incidence of these fevers in China depends on the effective prevention and control actions in such hotspot regions.

The grey model and the SARIMA model, two commonly applied statistical models to fit and predict the incidence of infectious diseases, were applied in parallel on typhoid and paratyphoid in this study. Based on the grey theory, GM (1,1) model constructed a new cumulative time series from the original one, and the trends of new series could be approximated by the solution of the first-order linear differential equation [36]. Unlike some parametric models requiring large sample size and unfounded assumption of variable distributions, GM (1,1) model needs a small sample size to achieve high prediction accuracy, and is mainly distribution-independent [37]. However, the establishment of grey model requires non-fluctuating data, so GM (1,1) is not suitable for the prediction of monthly cumulative incidence with seasonal pattern. In comparison, the SARIMA model fitted the seasonal periodicity and long-term trend of time series, and iterative comparison of parameter settings would fine-tune and select the optimal model [38, 39]. The final SARIMA model fitted the long-term trend and annual seasonality for typhoid and paratyphoid fevers both for the available time range and for the near future.

To the best of our knowledge, this is the first study to explore in detail factors of age groups and regional distribution for the cumulative incidence of typhoid and paratyphoid fevers in China, and apart from descriptive analysis, statistical learning models of both GM (1,1) and SARIMA are applied to formulate the underlying incidence pattern as well as to predict the trend in a prospective manner. Such methods benefit from epidemiology-based retrospective databank and result in evidence-driven knowledge for the prevention and control of both fevers: with a general drop of incidence in recent years, typhoid and paratyphoid remain risky infectious diseases in China, and should be closely monitored as potential threats to the public health particularly in the summer season and in the high-risk southwestern regions (most notably Yunnan province).

## Supporting information

S1 Fig Cumulative incidences of typhoid and paratyphoid from 2004 to 2016 in 31 provincial regions. (DOCX)

S2 Fig Geographical map of China. (DOCX)

S3 Fig Cumulative incidences of typhoid and paratyphoid in four segments of China. Note: 31 provinces were divided into 4 segments as following: Southwest (Sichuan, Chongqing, Tibet, Guangxi, Guizhou, Yunnan), Northwest (Shaanxi, Qinghai, Shanxi, Xinjiang, Ningxia, Inner Mongolia, Gansu), Northeast (Heilongjiang, Jilin, Liaoning, Beijing, Tianjin, Hebei, Anhui, Jiangsu, Shandong, Henan), and Southeast (Shanghai, Zhejiang, Jiangxi, Fujian, Hunan, Hubei, Guangdong, Hainan).
(DOCX)

S1 Table Comparison of actual and predicted incidence of typhoid and paratyphoid fevers by GM (1,1). (DOCX)

S2 Table Comparison of actual and predicted incidence of typhoid and paratyphoid fevers in 2016 by SARIMA (0,1,7) × (1,0,1) _12_. (DOCX)
