# Supplementary figures and images for: Time series analysis of cumulative incidences of typhoid and paratyphoid fevers in China using both Grey and SARIMA models
Source: PLoS One. 2020 Oct 28;15(10):e0241217. doi: 10.1371/journal.pone.0241217 (PMC7592733; doi:10.1371/journal.pone.0241217)

**S1 Fig**


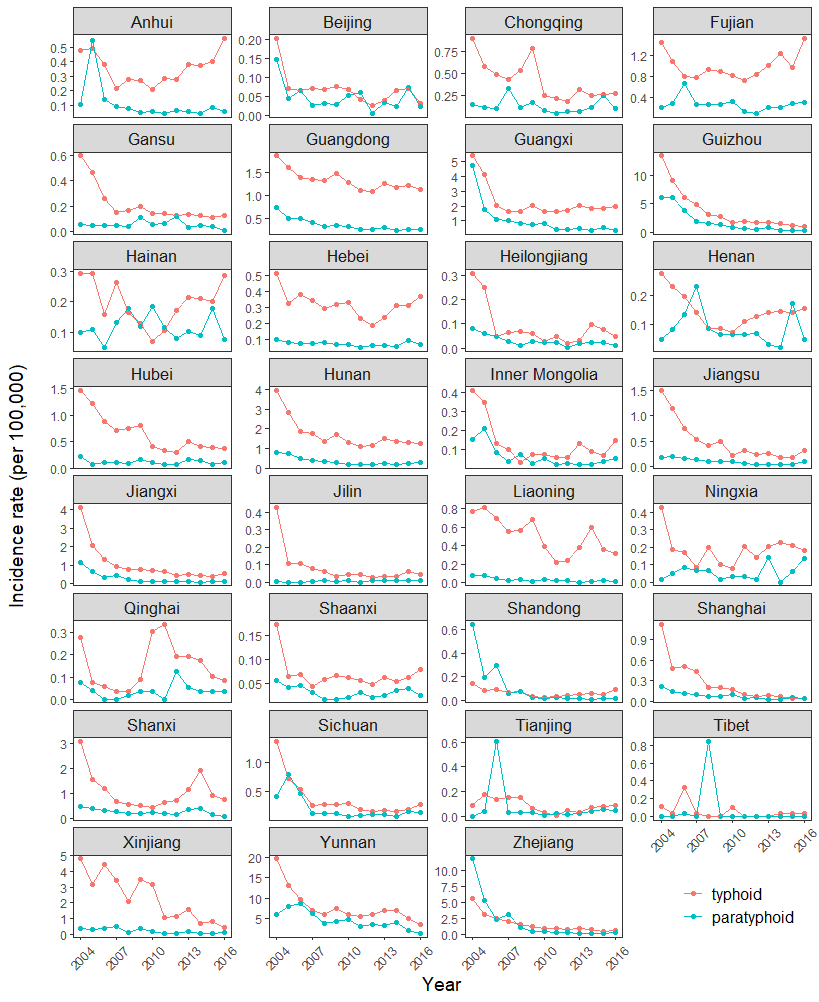

Supplement: S1 Fig — (DOCX) [file pone.0241217.s001.docx]

**S2 Fig**


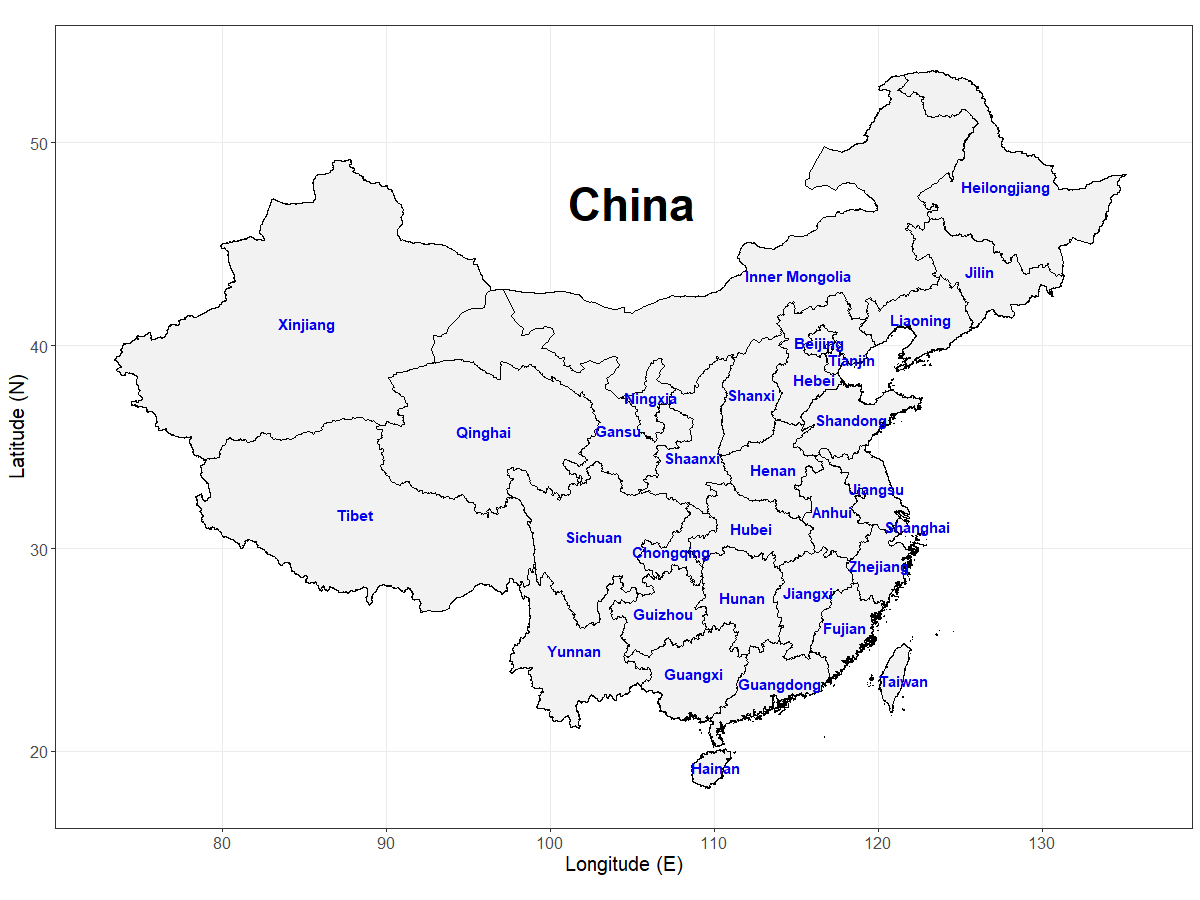

Supplement: S2 Fig — (DOCX) [file pone.0241217.s002.docx]

**S3 Fig**


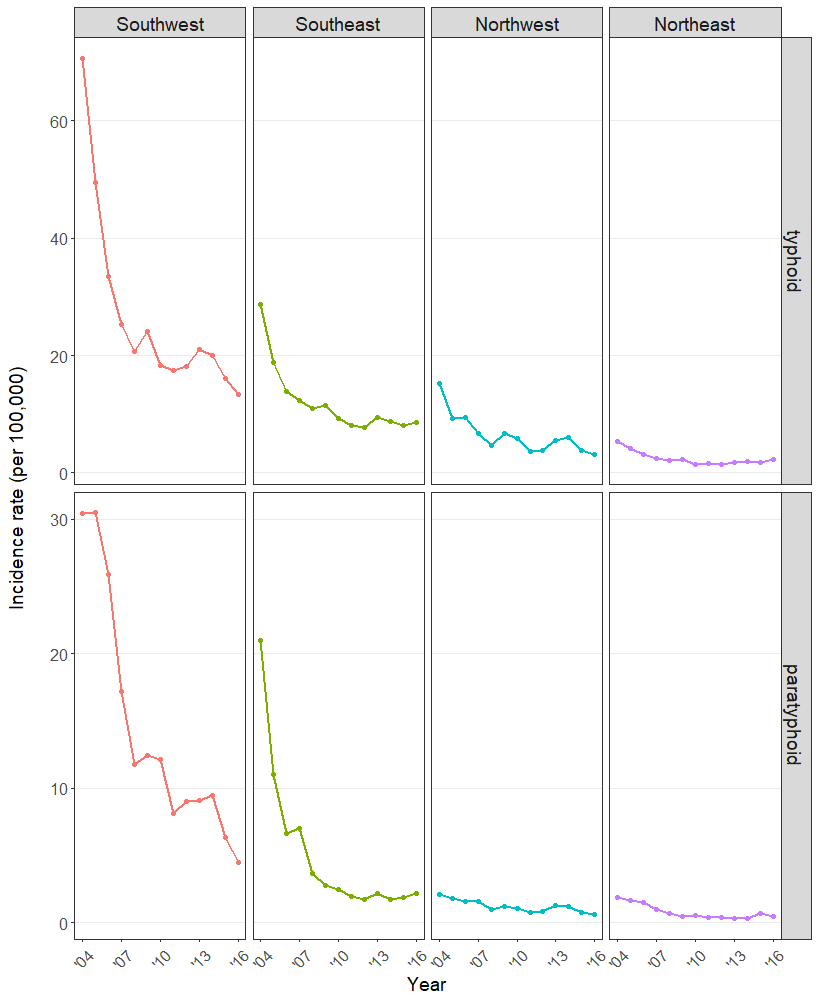

Supplement: S3 Fig — Note: 31 provinces were divided into 4 segments as following: Southwest (Sichuan, Chongqing, Tibet, Guangxi, Guizhou, Yunnan), Northwest (Shaanxi, Qinghai, Shanxi, Xinjiang, Ningxia, Inner Mongolia, Gansu), Northeast (Heilongjiang, Jilin, Liaoning, Beijing, Tianjin, Hebei, Anhui, Jiangsu, Shandong, Henan), and Southeast (Shanghai, Zhejiang, Jiangxi, Fujian, Hunan, Hubei, Guangdong, Hainan). (DOCX) [file pone.0241217.s003.docx]
