# Supplementary material for: Time series analysis of cumulative incidences of typhoid and paratyphoid fevers in China using both Grey and SARIMA models
Source: PLoS One. 2020 Oct 28;15(10):e0241217. doi: 10.1371/journal.pone.0241217 (PMC7592733; doi:10.1371/journal.pone.0241217)
Supplement: S1 Table — (DOCX) [file pone.0241217.s004.docx]

**S1 Table. Comparison of actual and predicted incidence of typhoid and paratyphoid fevers by GM (1,1)**

| Year | typhoid | |  | paratyphoid | |
| --- | --- | --- | --- | --- | --- |
|  | Actual incidence | Predicted incidence |  | Actual incidence | Predicted incidence |
| 2004 | 2.54 | 2.54 |  | 1.26 | 1.26 |
| 2005 | 1.71 | 1.38 |  | 0.96 | 0.86 |
| 2006 | 1.25 | 1.26 |  | 0.74 | 0.71 |
| 2007 | 0.98 | 1.15 |  | 0.57 | 0.59 |
| 2008 | 0.83 | 1.05 |  | 0.36 | 0.49 |
| 2009 | 0.94 | 0.96 |  | 0.34 | 0.41 |
| 2010 | 0.73 | 0.87 |  | 0.32 | 0.34 |
| 2011 | 0.65 | 0.80 |  | 0.23 | 0.28 |
| 2012 | 0.65 | 0.73 |  | 0.24 | 0.23 |
| 2013 | 0.79 | 0.66 |  | 0.25 | 0.20 |
| 2014 | 0.77 | 0.61 |  | 0.24 | 0.16 |
| 2015 | 0.65 | 0.55 |  | 0.21 | 0.13 |
| 2016 | 0.63 | 0.49 |  | 0.17 | 0.11 |
| 2017* |  | 0.43 |  |  | 0.09 |
| 2018* |  | 0.39 |  |  | 0.07 |

Note: cumulative incidence as incidence per 100,000 people. * data for 2017 and 2018 were not individually available in the public health database, but the combined data were reported by official government news releases.
