# Supplementary material for: Time series analysis of cumulative incidences of typhoid and paratyphoid fevers in China using both Grey and SARIMA models
Source: PLoS One. 2020 Oct 28;15(10):e0241217. doi: 10.1371/journal.pone.0241217 (PMC7592733; doi:10.1371/journal.pone.0241217)
Supplement: S2 Table — (DOCX) [file pone.0241217.s005.docx]

**S2 Table. Comparison of actual and predicted incidence of typhoid and paratyphoid fevers in 2016 by SARIMA (0,1,7) × (1,0,1)** **_12_**

| Month | typhoid | |  | paratyphoid | |
| --- | --- | --- | --- | --- | --- |
|  | Actual incidence | Predicted incidence |  | Actual incidence | Predicted incidence |
| Jan 16 | 0.0344 | 0.0450 |  | 0.0093 | 0.0120 |
| Feb 16 | 0.0362 | 0.0383 |  | 0.0093 | 0.0082 |
| Mar 16 | 0.0527 | 0.0484 |  | 0.0115 | 0.0117 |
| Apr 16 | 0.0512 | 0.0493 |  | 0.0139 | 0.0138 |
| May 16 | 0.0568 | 0.0615 |  | 0.0171 | 0.0184 |
| Jun 16 | 0.0598 | 0.0708 |  | 0.0187 | 0.0234 |
| Jul 16 | 0.0682 | 0.0715 |  | 0.0185 | 0.0232 |
| Aug 16 | 0.0726 | 0.0660 |  | 0.0209 | 0.0259 |
| Sep 16 | 0.0558 | 0.0608 |  | 0.0168 | 0.0251 |
| Oct 16 | 0.0552 | 0.0560 |  | 0.0126 | 0.0189 |
| Nov 16 | 0.0441 | 0.0454 |  | 0.0101 | 0.0136 |
| Dec 16 | 0.0395 | 0.0397 |  | 0.0098 | 0.0096 |
|  |  |  |  |  |  |

Note: cumulative incidence as incidence per 100,000 people.
